# Editorial: Cancer treatment and early detection targeting HER receptors, Volume II

**DOI:** 10.3389/fmolb.2023.1229765

**Published:** 2023-06-22

**Authors:** Xiaoqing Cai, Libing Zhang, Shengxi Chen

**Affiliations:** ^1^ School of Pharmaceutical Sciences, Sun Yat-sen University, Guangzhou, China; ^2^ Tianjin Key Laboratory of Molecular Optoelectronic, Department of Chemistry, Tianjin University, Tianjin, China; ^3^ Biodesign Center for Bioenergetics, Arizona State University, Tempe, AZ, United States

**Keywords:** cancer targeted therapy, early detection, EGFR, HER family, HER2, HER3

HER receptors play important roles in the process of initiation, development, migration, and invasion of many types of cancers. Homodimerization or heterodimerization of HER receptors activates their downstream pathways. Inappropriate activation of HER1, HER2 or HER3 usually leads to tumor progression. However, the role of HER4 in cancer progression has conflicting data in different studies (El-Gamal et al., 2021).

To date, a number of target-therapy agents have been approved by the FDA to treat the HER(s) overexpressing cancer patients. Four anti-HER1 antibodies (amivantamab, cetuximab, necitumumab, and panitumumab) and eight small molecule drugs (afatinib, dacomitinib, erlotinib, gefitinib, lapatinib, mobocertinib, neratinib, and osimertinib) have been approved to treat HER1 positive cancers (Oh and Bang, 2020; Rosenkranz and Slastnikova, 2020; Olivier and Prasad, 2022). Among these four HER receptors, HER2 is another remarkable target for drug development. Five HER2-targeting monoclonal antibodies or antibody-drug conjugates (trastuzumab, pertuzumab, margetuximab, ado-trastuzumab emtansine and fam-trastuzumab deruxtecan) and one HER2 specific small molecule drugs (tucatinib) have been approved by the FDA and more novel HER2 target agents are continuing to be developed (Zhang et al., 2019; Oh and Bang, 2020; Zhang et al., 2020). Lapatinib targeting both HER1 and HER2 and neratinib irreversibly binding to HER1, HER2 and HER4 have been approved to treat breast cancer (Oh and Bang, 2020). In recent years, HER3 has gained more attention as it is an essential heterodimeric partner for HER1 and HER2, which has the potential to involve drug resistance to anti-HER1 and anti-HER2 therapies. Recently, one HER3-targeted antibody-drug conjugate (patritumab deruxtecan) has been approved for the treatment of HER1-mutant non-small cell lung cancer (NSCLC) patients (Cai et al., 2022). Even though the target-therapy agents for HER1–3 have been successfully developed, there are still challenges to overcoming the drug resistance and lacking efficacy in HER-low patients.

In this Research Topic, Nizioł et al. investigated the proliferative capacity of recombinant human prolidase (rhPEPD) in human HaCaT keratinocytes (Nizioł et al.). The results showed that both active and inactive rhPEPD activate HER1-dependent cell growth. To develop new HER2-target agents, Moghadam computationally engineered a HER2-dependant penetrating peptide to the chain A of ricin which is responsible for the ribosome inactivation (Ahmadi Moghadam et al.). The results showed that two chimeric proteins could bind the receptor with the greatest affinity. In addition, Zheng et al. reviewed the therapeutic role of a novel antibody-drug conjugate, Ado-trastuzumab emtansine (T-DM1) targeting HER2-positive cancers (Zheng et al.). These cancers include gastric cancer, non-small cell lung cancer (NSCLC), and colorectal cancer.

Different cancers have different levels of HER receptors. To guide the precision therapy for each cancer patient using current approved target-therapy agents, early detection and quantification of each HER receptor are necessary for assessment, treatment, and prognosis. In the clinic, current standard HER detection methods include fluorescence *in situ* hybridization (FISH) assays to detect HER DNAs; immunohistochemistry (IHC) assays to detect HER proteins; and RNA *in situ* hybridization (RNA-ISH) assays to detect HER RNAs.

**FIGURE 1:**
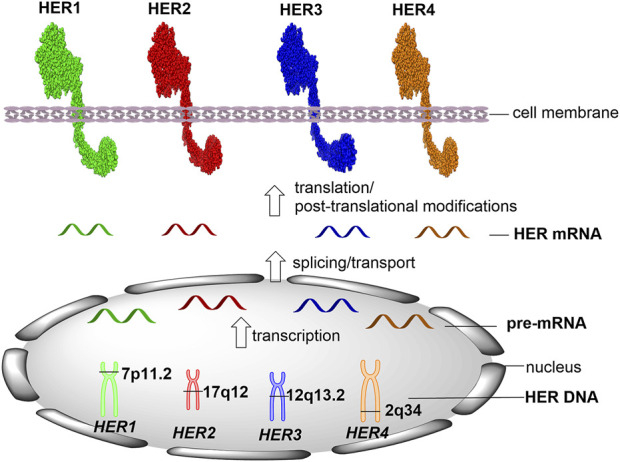
Location of HER targets (DNAs, RNAs and proteins) in human cells.

Current gold standard methods for HER detection and quantification are carried out using formalin-fixed paraffin-embedded (FFPE) tissue specimens. It is important to clearly identify the position of each target in human cells. *HER1* (also known as ERBB1/EGFR) gene locates on the short arm of chromosome 7 (7p11.2), which contains 192,612 bases of DNA on the plus strand (Figure 1, GeneCards, The Human Gene Database). Following the transcription and pre-mRNA splicing, the mature HER1 mRNA is transported into the cell cytoplasm and translated into HER1 protein that contains 1,210 amino acids. *HER2* (ERBB2) gene locates on the long arm of chromosome 17 (17q12), which is encoded by 42,513 bases of DNA on the plus strand and translated into 1,255 amino acids. *HER3* (ERBB3) gene locates on chromosome 12 at the long arm (12q13.2), which contains 26,707 bases of DNA on the plus strand and translates into HER3 protein containing 1,342 amino acids. *HER4* (ERBB4) gene locates on the long arm of chromosome 2 (2q34), which is encoded by 1,163,125 bases of DNA on the minus strand and translates into 1,308 amino acids.

Recently, the treatment for HER2-positive cancers is more complicated than other HER-targeting treatments, especially since trastuzumab deruxtecan (T-DXd) was approved by FDA to treat HER2-low metastatic breast cancer (U.S. FDA, 2022). In this special Research Topic, Sajjadi et al. discussed practical solutions that could enhance HER-low assessment and provided an overview of the existing HER2-low identification in breast cancer (Sajjadi et al.).

The FFPE cancer tissue samples for *in situ* detection and quantification of HER status are obtained from the primary tumor. The results can be used to guide treatment decisions and predict patient outcomes. However, most of the tumors have a high risk of drug resistance and recurrence after HER-target therapy because of dynamic changes in HER levels. Therefore, it is important to develop a real-time detection method for monitoring the HER levels during and after the target treatment. In this Research Topic, Miladinova reviewed HER-2 specific positron emission tomography (PET) imaging to test the HER receptors *in vivo*, in which a variety of molecular probes are used to label HER2 receptors for imaging within the living body (Miladinova). The PET imaging method provided important information about the spatial distribution and intensity of HER2 proteins in tumors.

In summary, this special Research Topic provides updated development of cancer therapeutics targeting HER receptors. It also gives interesting insights into new and complementary approaches for the detection and quantification of HER receptors.
